# Characterization of Nematicidal Activity and Nematode-Toxic Metabolites of a Soilborne *Brevundimonas bullata* Isolate

**DOI:** 10.3390/pathogens11060708

**Published:** 2022-06-20

**Authors:** Jiaoqing Li, Meijuan Ding, Xiaowen Sun, Zhe Li, Liangzheng Xu, Lin Li

**Affiliations:** 1 Guangdong Provincial Key Laboratory of Conservation and Precision Utilization of Characteristic Agricultural Resources in Mountainous Areas, School of Life Sciences, Jiaying University, Meizhou 514015, China; lijiaoqing@jyu.edu.cn; 2 State Key Laboratory of Agricultural Microbiology, Huazhong Agricultural University, Wuhan 430070, China; dingmeijuan@webmail.hzau.edu.cn (M.D.); sunxiaowen525@webmail.hzau.edu.cn (X.S.); lizhe9913@163.com (Z.L.)

**Keywords:** nematicidal bacteria, screening and characterization, *Brevundimonas bullata*, *Meloidogyne incognita*, *Caenorhabditis elegans*

## Abstract

The increasing prevalence of crop-threatening root-knot nematodes (RKNs) has stimulated extensive research to discover effective nematicides. A highly focused strategy for accomplishing this is the development of biocontrol agents by a variety of soilborne microorganisms, as different bacterial metabolites have demonstrated promising nematicidal activities. In this study, we characterized the nematicidal and suppressive activity of a bacterial isolate against the agriculturally important RKN *Meloidogyne incognita* and the model nematode *Caenorhabditis elegans*, and the main *M. incognita*-toxic metabolite of the strain. After a preliminary screening of 22 bacterial isolates with a corrected mortality (CM) of whole-cell culture greater than 50% against *C. elegans* from different RKN-incident soils in China, a total of 14 isolates with CM of the supernatant of culture suspension (SCS) higher than 50% against both *M. incognita* and *C. elegans* were rescreened. An isolate with the highest CM of 86.1% and 95.0% for *M. incognita* and *C. elegans*, respectively, was further identified as the species *Brevundimonas bullata* via morphological examination, physiological and biochemical assays and alignment analysis of 16S rRNA gene sequences. The SCS of this strain, namely, *B. bullata* MB756, exhibited synchronous *M. incognita* killing activity along with significant detrimental effects on the growth, brood size, and locomotion of *C. elegans*. The effects of heat treatment, pH, inoculations, and protease K proteolysis on the CM of MB756 SCS were evaluated. A major *M. incognita*-toxic substance in the MB756 SCS was assayed and identified using thin-layer chromatography, column chromatography and high-performance liquid chromatography with a mass spectrometer, and it was preliminarily identified as 2-ethylhexan-1-ol, with a molecular formula of C_8_H_18_O and a molecular weight of 130.3 Da.

## 1. Introduction

Plant parasitic nematodes (PPNs) are a large group of plant pathogens belonging to the phylum Nematoda that cause an annual global loss greater than 157 billion USD [1]. The main agriculturally important PPNs are root-knot nematodes (RKNs), which dominate the genus *Meloidogyne*, with more than 100 species that inflict serious damage by infecting the roots of a wide range of host plants [2,3]. In China, RKNs are found in more than 27 provinces and cities, harming more than 3000 types of plants, and the annual loss caused by vegetable cultivation alone has accounted for 500 million USD [4]. Of the various soil-transmitted RKNs, the most prevalent RKN, *Meloidogyne incognita*, is widely distributed throughout vast areas across the temperate and tropical zones of northern and southern China and causes serious damage each year to vegetables, fruits, medicinal plants, and garden trees nationwide [5].

Various strategies have been implemented to control *M. incognita*. Conventional approaches include chemical nematicides, physical and agricultural planting efforts, and the planting of nematode-resistant plant varieties [6]. Although chemical agents are still irreplaceable as a typical means of controlling *M. incognita* and other harmful nematodes, increasing concern regarding human and environmental safety and the expanding concept of green and healthy farming has restricted the use of these nematicides. Alternatively, there is an increasing incentive for developing bionematicides, which have been characterized by advantageous properties, such as long-term control, environmental friendliness, and difficult resistance development for target nematodes [7]. Thus, the R&D of bacterial nematicides with specific *M. incognita*-toxic substances has received increasing attention [8,9].

Bacterial nematicides refer to a variety of biocontrol agents that use live bacterial cells themselves and/or their primary (or secondary) metabolites to reduce or control the worm population density and the impact of PPNs [10]. In recent years, many bacteria have been confirmed to have promising nematicidal activity. These bacteria include *Bacillus subtilis* [11], *Bacillus thuringiensis* [12,13], *Bacillus amyloliquefaciens* [14], *Bacillus cereus* [15], *Bacillus firmus* [16,17,18], *Bacillus nematocida* [19], *Pseudomonas aeruginosa* [20,21,22], *Pseudomonas putida* [23], *Pseudomonas simiae* [24], *Pseudomonas syringae* [25,26,27], *Burkholderia cepacia* [28], *Streptomyces avermitilis* [29,30], and *Pasteuria penetrans* [31]. These nematicidal bacteria affect the target nematodes through different mechanisms, such as restraining the growth and reproduction of nematodes and direct pathogenesis, which are carried out by the production of toxic proteins [32,33], enzymes [12,26,34], small-molecule metabolites [23,24,35], or invasive parasitization [36,37]. In this respect, bacterial nematicides have shown particular usefulness in terms of their relatively high specificity, their ability to act on the underground parts of plants, and the difficulty in resistance-development in nematodes. However, nematicidal bacteria with high nematicidal activity and market competitiveness are still extremely lacking relative to the large market demand to date. Therefore, it is necessary to search for new nematicidal bacteria as well as highly nematicidal substances with market potential.

Soilborne microorganisms are closely related to plant roots and rhizosphere nematodes, which constitute a complicated and dynamically interinhibitive microecosystem. Therefore, screening nematicidal microorganisms from *M. incognita*-incident soils can be an effective and applicable approach for developing bionematicides to prevent the plant damage caused by *M. incognita*. In the current study, we performed a joint two-round screening of culturable nematicidal bacteria from RKN-incident soils that were collected from different areas of China, with a particular focus on those where the supernatant of the culture suspension (SCS) was toxic to *M. incognita*. After a preliminary screening process from whole-cell suspension using the model organism *Caenorhabditis elegans* as the target nematode, which has been proven to be effective and time-saving in terms of facile cultivation processes and the short lifecycle for facilitating bioassays [24,38], the resultant bacterial isolates were rescreened to evaluate the nematicidal activity of their SCS against both *C. elegans* and *M. incognita*. Ultimately, an isolate was obtained and identified as *Brevundimonas bullata* (named MB756) through morphological examination, partial physiological and biochemical assays, and alignment analysis of its 16S rRNA gene sequences. This strain was subjected to further assays of nematicidal activity at different growth phases, of suppressive effects on the growth, brood size, and locomotion of *C. elegans*, and of the effects of heat treatment, pH, inoculations, and protease K proteolysis on nematicidal activity. A major *M. incognita*-toxic substance was assayed and preliminarily identified using thin-layer chromatography (TLC), silica gel column chromatography (SGCC), and high-performance liquid chromatography with a mass spectrometer (LC–MS), with the aim of isolating and identifying highly *M. incognita*-toxic bacterial strains and pursuing the development of metabolite-based bionematicides.

## 2. Results

### 2.1. Screening and Identification of the Highly Nematicidal Brevundimonas Bullata MB756 and Its Nematicidal Efficacy

Twenty-six soil samples were collected from severe RKN-infected soils planted with vegetables (mainly tomatoes and cucumbers) and fruits (mainly kiwifruit and grapes) in China. A total of 232 culturable bacterial strains with rapid growth activity (i.e., visible single colonies approximately 3 mm in diameter in 24 h cultured on NA plates) were initially collected after enrichment culturing and streaking on plates to yield pure colonies. For the first-round screening of nematicidal bacteria, these isolates were cultured in NBM broth for 24 h, the cell suspension of each isolate was then bioassayed against *C. elegans*, and 22 isolates among them with a corrected mortality over 50% were selected. Subsequently, the supernatant of culture suspension (SCS) after 24 h of culture in NBM broth of all 22 isolates was rescreened using *M. incognita* and *C. elegans* as the target nematodes, yielding 14 strains with higher corrected mortalities against both nematodes (Supplementary Figure S1). One of these isolates (No. 5-7) exhibited the highest corrected mortality toward *M.* *incognita* and *C. elegans* by 86.1% and 95.0%, respectively, and was selected for follow-up studies.

**Figure 1:**
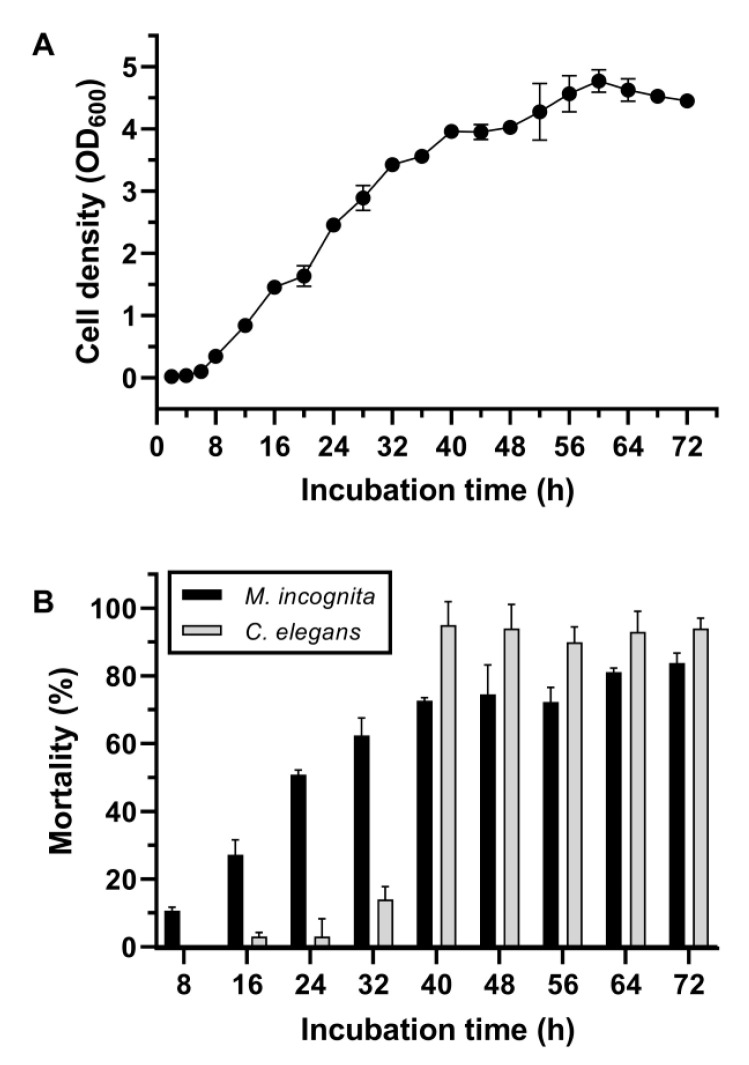
Growth curve (**A**) and nematicidal bioassays of the SCSs under different incubation times (**B**) of isolate No. 5-7. In (**A**) and (**B**), the same batch of the cell suspension culture was synchronously used for the growth curve measurement and the corrected mortality bioassay. Prior to the bioassay, each prepared SCS sample was adjusted to a pH of 7 and then filtered through a 0.22-μm microporous membrane to remove cells or debris. After an incubation at 20 °C for 5 days, larvae were examined for alive/dead status using a platinum wire. Error bars represent the standard deviations from the mean of three independent experiments.

The isolate No. 5-7 was subjected to the assays of cell growth and the corrected mortality against *M. incognita* and *C. elegans* at different growth phases. Figure 1A shows a fast growth profile of this isolate under normal culturing conditions by entering the logarithmic growth phase at approximately 6 h, the stationary phase at approximately 40 h, and the decline phase at approximately 60 h. Monitoring of the corrected mortality of isolate No. 5-7 against *M. incognita* showed a fast and steadily increasing pattern during the culture time-course, suggesting the constitutive production of toxic substances by *M.* *incognita* along with synchronous cell growth. In contrast, the substantially corrected mortality against *C. elegans* appeared in the stationary growth phase and then maintained high levels across the rest of the culture time course (Figure 1B). Therefore, these results suggest in principle that the toxic substances against *M. incognita* and *C. elegans* were different and were synthesized at different growth phases by this strain.

**Figure 2:**
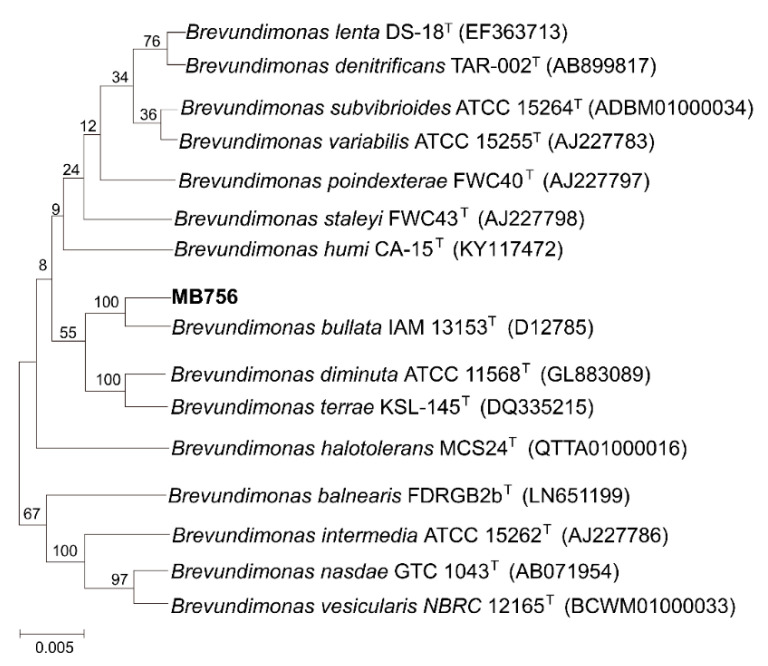
Neighbor-joining tree generated using MEGA 5 on the basis of 16S rRNA gene sequences of isolate No. 5-7. Bootstrap values are shown as percentages of 1000 replicates when these values were greater than 50%. The scale bar represents 0.5% substitution per nucleotide position.

The partial morphological, cultural, physiological, and biochemical characteristics of isolate No. 5-7 are shown in Supplementary Figure S2. Briefly, the colonies of isolate No. 5-7 on LB plates were circular, milky white, smooth, slightly convex, and brown-yellow, with moist and opaque surfaces, and were easy to pick up. Cells are Gram-negative and rod-shaped and occur singly, with no spore formation. The indole production, V.P. and M.R. tests, H_2_S production, gelatin and starch hydrolysis, and phenylalanine dehydrogenase were negative, whereas oxidase, catalase, and citrate utilization were positive. All these phenotypic characteristics and the DNA (G+C) mol% of approximately 67.6% are consistent with the description of the species *Brevundimonas bullata* [39]. Furthermore, as shown in Figure 2, the phylogenetic analyses based on 16S rRNA gene sequences revealed that isolate No. 5-7 fell into the closest clade with the species *B.* *bullata* IAM 13153^T^, with both strains sharing 99.93% sequence identity of their 16S rRNA genes. Based on these results, isolate No. 5-7 was preliminarily identified as *B. bullata* and named MB756.

### 2.2. Effects of SCS of MB756 Cells on the Growth, Brood Size, and Mobility of C. elegans

**Figure 3:**
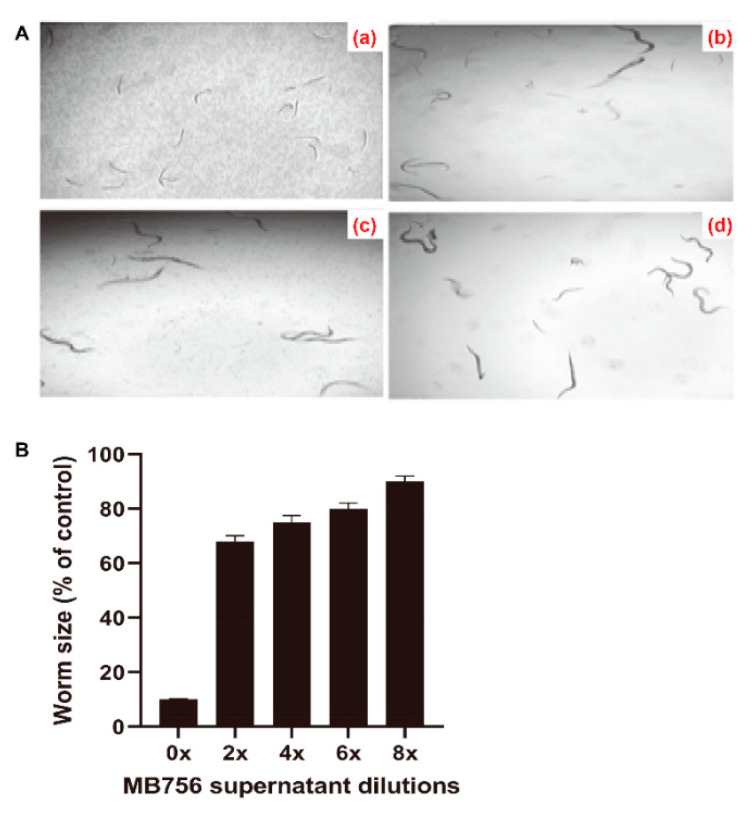
Inhibitory effect of MB756 culture supernatant on the growth of *C. elegans* L1 larvae. (**A**), Morphology of *C. elegans* larvae after treatment with 0 (**a**), 2× (**b**), and 8× (**c**) diluted MB756 culture supernatants for 3 days. Larvae in LB broth (**d**) were used as the negative control; (**B**), Growth assay of *C. elegans* treated with gradient doses of MB756 culture supernatant for 3 days. Error bars represent the standard deviations from the means of three independent experiments.

To investigate the effects of the SCS of MB756 on the growth of *C. elegans*, the SCS stock solution after 60 h of incubation was diluted to 0, 2×, 4×, 6×, and 8× diluted concentrations and was used for bioassays against L1-stage larvae of *C. elegans*. Figure 3A shows that the SCS stock caused a substantial suppressive effect on the growth of the larvae, as a nearly negative growth pattern was visualized after treatment for 3 days (Figure 3Aa), whereas other diluted SCS solutions (2× and 8×, Figure 3Ab,Ac) caused only slight suppression compared with the negative control (Figure 3Ad). These phenotypes were consistent with the measured worm length after treatment with different dilutions. As shown in Figure 3B, the average length of larvae treated with the SCS stock solution for 3 days accounted for only approximately 10% of the control, which strongly contrasted with approximately 68% to 90% of the control by other dilutions.

**Figure 4:**
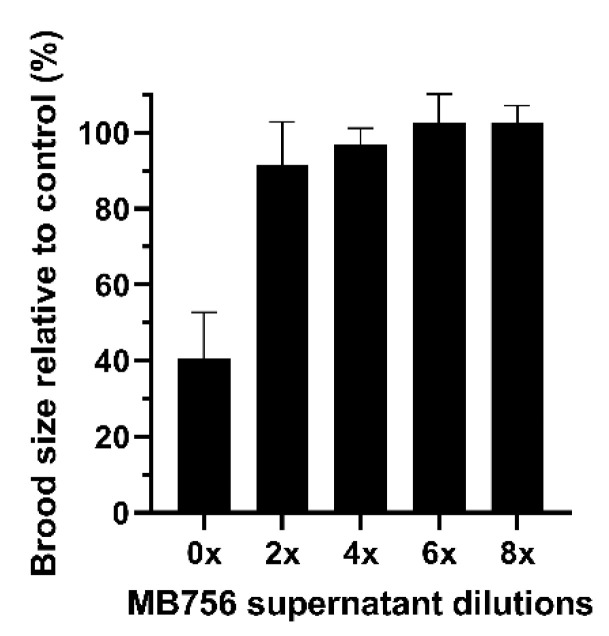
Brood size assay of *C. elegans* L4 larvae using different diluted MB756 culture supernatants. The assay was conducted for 3 days. Error bars represent the standard deviations from the mean of three independent measurements.

Figure 4 shows that the SCS stock solution also caused significant suppression of brood size. After treating the larvae for 3 days, only approximately 40% of the brood size was retained compared with the slight effects of other dilutions.

**Figure 5:**
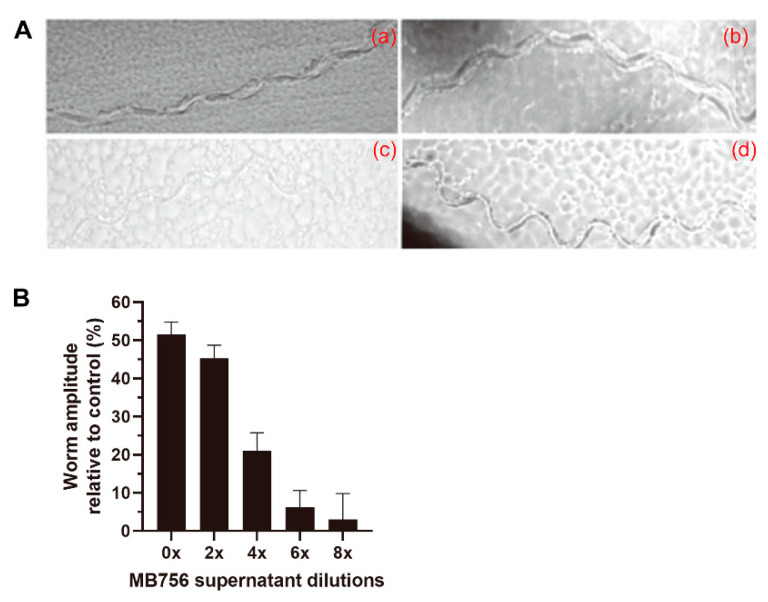
Effect of MB756 culture supernatant on *C. elegans* motility. (**A**), Track patterns of larvae on NGM plates with *C. elegans* L4 larvae treated with 0× (**a**), 2× (**b**), and 8× (**c**) diluted MB756 culture supernatant for 24 h. Larvae treated with only NGM medium were used as the negative control (**d**). (**B**), The calculated amplitude values of the worm tract under different dilutions of MB756 culture supernatant. Six larva individuals were assayed in each experiment. Error bars represent the standard deviations from the mean of three independent experiments.

To investigate the effect of different dilutions of the MB756 SCSs on the motility of *C. elegans*, *Escherichia coli* OP50 was spread on NGM plates, and the L4-stage *C. elegans* larvae treated with various diluted SCSs were placed on the plates to examine their mobile track patterns and amplitude extent under a microscope. Figure 5 shows that while the larvae exhibited a near “S”-shape track for the negative control (Figure 5Ad), the track of the larvae under the treatment of the SCS stock solution appeared to be straight (Figure 5Aa), and the amplitude of these larvae was reduced by approximately 45% compared with the LB control (Figure 5B).

### 2.3. Effects of Different Temperatures, pH Values, Inoculation Times, and Proteolysis Hydrolysis Treatments on the Nematicidal Activity of the MB756 SCS against M. incognita

**Figure 6:**
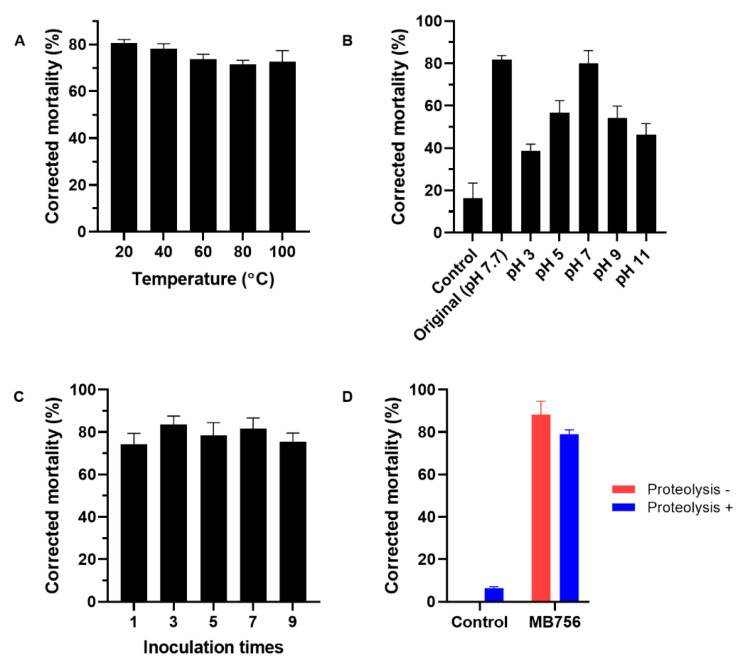
Effects of different temperatures (**A**), pH values (**B**), inoculation times (**C**), and proteolysis treatment (**D**) on the nematicidal activity of MB756 culture supernatant against *M. incognita*. Error bars represent the standard deviations from the mean of three independent experiments.

To evaluate the thermostability and pH adaptability range of the MB756 SCS, the MB756 SCS that was filtered through a 0.22-μm microporous membrane was incubated at 20 °C, 40 °C, 60 °C, 80 °C, and 100 °C in a water bath for 30 min or was adjusted to a series of pH values of 3-11 and then subjected to bioassays against *M. incognita*. Figure 6A shows that only a slight reduction of *M. incognita* mortality by approximately 5% was seen under the heat treatments across the range of 20 °C to 100 °C, suggesting the strong thermostability of *M. incognita*-toxic substances in MB756 SCS. Figure 6B shows that the highest mortality of the MB756 SCS was recorded at pH 7, which was close to the SCS stock solution (pH 7.7), whereas at pH 3 and pH 11, the activity was reduced to approximately 38% to 56% compared with that of the SCS stock without pH adjustment, suggesting a relatively wide pH adaptability range of the MB756 SCS in terms of *M. incognita* mortality.

To investigate the effect of the inoculation times of MB756 on *M. incognita* mortality, MB756 was inoculated successively 7 times on the plates, and the SCS from the 1st, 3rd, 5th, 7th, and 9th inoculations were bioassayed against *M. incognita*. Figure 6C shows no significant effect of these inoculations on *M. incognita* mortality, reflecting the genetic stability of the production of the active substances following successive inoculations, which is considered to indicate the potential for industrial large-scale production. Moreover, protease K proteolysis on the MB756 SCS also did not cause significant loss of *M. incognita* mortality, suggesting in principle that the main nematicidal substance was nonproteinic in nature.

### 2.4. Purification and Preliminary Identification of the Active Substances in the MB756 SCS

To investigate the organic polarity and extractability of the nematicidal active substances in the MB756 SCS, several common organic solvents, such as petroleum ether, chloroform, ethyl acetate, and n-butanol, were used as the organic phases to extract the active substances from the MB756 SCS. Table 1 shows that the fraction extracted from ethyl acetate maintained a high *M. incognita* mortality by approximately 85%, which was close to that of the MB756 stock solution and was much higher than that of other extracted fractions. The petroleum ether phase also retained approximately 43% of the insecticidal activity. These results suggest that there appears to be a main nematicidal substance with a higher polarity and a subsidiary nematicidal substance with less polarity and a lower content in the MB756 SCS. Considering that ethyl acetate exhibited a good performance of the main nematicidal substances in the MB756 SCS, it was selected as the extraction solvent for the subsequent separation and purification of the *M. incognita*-toxic substances of the MB756 SCS.

**Figure 7:**
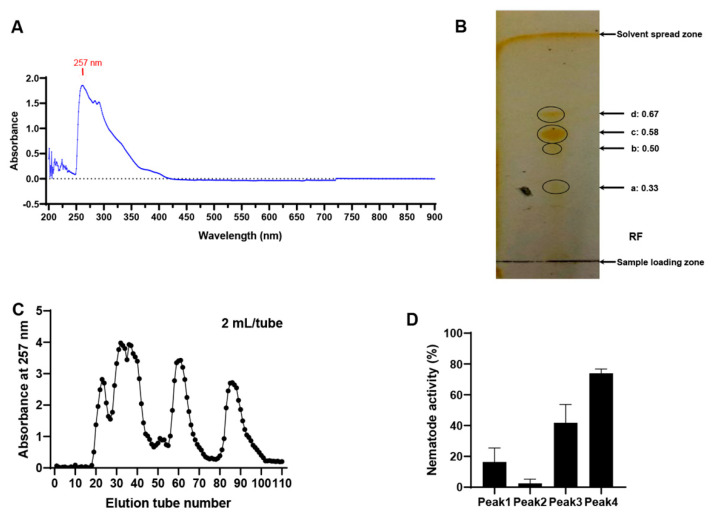
Full-wavelength adsorption scanning (**A**), TLC (**B**), SGCC (**C**), and bioassays against *M. incognita* (**D**) of the ethyl acetate extract of the MB756 SCS. TLC plate was fumigated with iodine vapor for color development.

Full-wavelength (190–900 nm) absorption spectrum scanning of the ethyl acetate extract of the MB756 SCS showed a maximum absorption peak at 257 nm (Figure 7A). TLC of the ethyl acetate extract of the MB756 SCS was performed using silica gel as the stationary phase. Several mixed solutions were compared for their separation efficiencies when used as a layer-developing solvent. These solutions included paired dichloromethane/methanol, chloroform/methanol, and petroleum ether/acetone at different ratios (volume/volume). An optimal mixed solvent pair was thus attributed to a 9:1 (vol/vol) mixture of chloroform and methanol. As shown in Figure 7B, 4 substance spots were visualized in the thin-layer chromatography profile without apparent tailing traces in each spot, suggesting the existence of at least 4 substances in the ethyl acetate phase, with *Rf* values of 0.33 (substance a), 0.50 (substance b), 0.58 (substance c), and 0.67 (substance d).

The ethyl acetate extract of the MB756 SCS was further separated by silica gel column chromatography (SGCC) using a mixed chloroform/methanol solvent (9:1, vol/vol) as the mobile phase and silica gel as the stationary phase, and the detection wavelength of column chromatography was set at 257 nm. A total of 4 main adsorption peaks of products were recorded from 4 sets of collected eluates in a fraction collector (Figure 7C), consistent with the TLC results (Figure 7B). These 4 collected fraction eluates were individually condensed via rotary evaporation to remove the solvents, and the solute substances were redissolved with double distilled water (ddH_2_O) and subjected to bioassays against *M. incognita*. Figure 7D shows that the solute substance from the 4th peak exhibited the highest *M. incognita* mortality of approximately 74%, whereas the others caused the mortality of *M. incognita* to be less than 40%. Therefore, these results suggest that the main *M. incognita*-toxic substance occurred in the 4th eluted peak.

### 2.5. Preliminary Identification of the Main M. incognita-Toxic Substance in the MB756SCS

**Figure 8:**
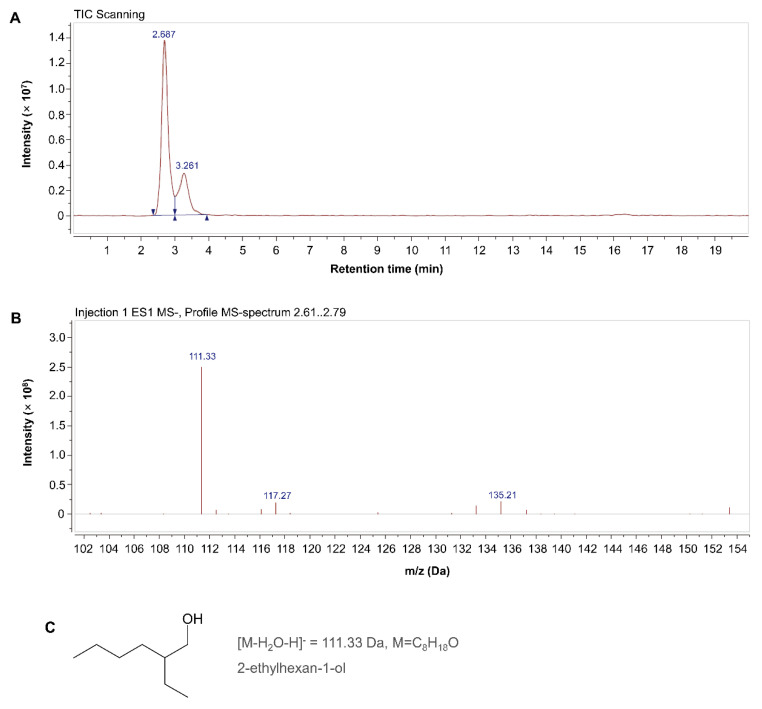
LC-MS analyses of the 4th eluted peak of the MB756 SCS for preliminary identification of the main nematicidal substance. (**A**), the scanning curve of total ion chromatogram (TIC); (**B**), mass spectrum peak 2.61–2.79 min; (**C**), structural formula of 2-ethylhexan-1.

The main *M. incognita*-toxic substance in the 4th eluted peak of the MB756 SCS was assayed using LC–MS. Figure 8A shows that there were two scanning peaks at retention times of 2.687 and 3.261 min in the liquid-phase TIC scanning profile. Subsequent ESI MS scanning in the spectrum region of 2.61–2.79 min was performed, yielding a mass peak spectrum at *m*/*z* 111.33 [M-H_2_O-H]^−^, which corresponds to a substance with a molecular formula of C_8_H_18_O and a relative Mw of 130.33 Da based on a search of a highly matched substance with the highest degree in the chemical database. ^1^H NMR (400 MHz, chloroform-d, ppm) chemical shifts of the compound were as follows: δ 3.51 (dd, *J* = 5.3, 1.6 Hz, 2H), 1.75 (s, 1H), 1.46–1.15 (m, 8H), 0.87 (td, *J* = 7.1, 3.0 Hz, 6H). This substance exhibits fundamental characteristics similar to those of a previously reported nematicidal substance by Zhang et al. [40], namely, 2-ethylhexan-1-ol. We are, therefore, preliminarily identifying this substance as 2-ethylhexan-1-ol, with the structural formula shown in Figure 8C.

## 3. Discussion

PPNs are a large variety of plant pests that cause serious damage to host plants, and RKNs have been recognized as the most destructive PPNs worldwide, seriously affecting the growth and development of many crops (e.g., soybeans, potatoes, wheat), vegetables (e.g., tomato, cucumber, eggplant), and various fruit trees, resulting in a significant reduction in crop yields and large economic losses each year. Due to the problems of pesticide residues, the difficulty of reaching the underground part of plants and relatively less reliance on the long-term pest management of chemical pesticides and biological control methods have attracted increasing attention. In this respect, a variety of nematicidal bacteria have received special attention due to their quick growth, strong tolerance to stressful environments, and ability to synthesize a wide variety of RKN-killing active substances. Currently, the most in-depth studies have been conducted on many RKN-killing bacteria, particularly those from *Bacillus* and *Pseudomonas*, such as *B. thuringiensis*, *B. subtilis*, and *B. cereus*, which have been successfully applied in the fields for biocontrol of RKNs during the cultivation of certain vegetables, including tomato, pepper, and cucumber, and have been listed as one of the most promising biocontrol agents [11,15,41,42,43]. In contrast, some *Pseudomonas* species are able to produce or secrete various enzymes [20,34], nematicidal substances [23,24], nematode-toxic metabolites [44], or multiple control mechanisms against RKNs, including the induction of systemic resistance (ISR). In this study, we selected an agriculturally significant RKN, the J2 larvae of *M. incognita,* as the target nematode pest with which to screen *M. incognita*-toxic bacterial isolates with highly active secretory metabolites from *M. incognita*-incident soils, resulting in the wild-type strain *B. bullata* MB756, which has been characterized to have highly dual nematicidal activity against both *M. incognita* and *C. elegans*. To the best of our knowledge, this is the first study to confirm the nematicidal activity of a *B. bullata* strain by producing a secretory metabolite pathogenic to the target nematode *M. incognita*.

Although a variety of RKN-pathogenic substances have been identified both intracellularly and extracellularly from different nematicidal bacteria [45,46], the current study mainly focused on the screening of RKN-killing substances from the extracellularly secretory metabolites of bacterial cultures, which are mainly based on the following considerations: (1) Currently, a variety of bacterial extracellular secretory nematicidal substances with application potential have been successfully characterized. Considering the simplification of the posttreatment process for formulation development and the saving of production costs, these extracellular active substances appear to be significantly more attractive. (2) Some small-molecule active substances secreted by cells have more R&D potential in terms of the effective control of RKNs such as *M. incognita*, not only because these small molecular substances often have greater resistance to protease proteolysis, as well as certain harsh environmental conditions such as acidic, alkali, and high-temperature conditions, which are more advantageous over proteinic substances in soils, but also because of the limitation of the entrance molecular size of the specialized feed stylet of PPN larvae, which only permits proteins of limited size and dimension to pass through [47,48] (e.g., ~28 kDa for the nematode *Heterodera schachtii* [49]); thus, the prevention and control of these RKNs by small molecular substances is technically feasible. Therefore, in this study, the cell-free supernatant of cell culture suspensions was used as the source for screening highly nematicidal substances. The screening results showed that from 232 culturable bacterial strains, 14 active strains were obtained with a mortality higher than 50% against both *C. elegans* and *M. incognita* (Figure S1), accounting for approximately 6% of the total initially tested strains, which included one strain with the highest mortality against both nematodes over 86%, indicating that this screening method is technically feasible.

Interestingly, the results of bioassays of the SCSs against *C. elegans* and *M. incognita* at different growth stages (Figure 1) showed that the incidence of their nematicidal activities against these two nematodes was not synchronous, reflecting the nematicidal activities possibly derived from different active substances that were expressed or synthesized at different stages of the cell life cycle. In view of the fact that *C. elegans* is only a general free-living nematode and *M. incognita* is a serious RKN, this study thereby focused on the isolation and identification of toxic substances to *M. incognita*, which was ultimately attributed to the small-molecule metabolite 2-ethylhexan-1-ol through continuous multipurification processing and preliminary identification. This substance exhibited several distinctive features, such as a high killing activity against *M.* *incognita* (Figure 1), strong thermal stability (Figure 6A), pronounced tolerance to protease proteolysis (Figure 6D), tolerance of acidic and alkali conditions (Figure 6B), and stable performance under continuous multiple-inoculation processing (Figure 6C), suggesting that this substance may have good potential for further development of a new *M. incognita*-controlling agent for agricultural use.

Various *Pseudomonas* strains have been characterized for their ability to produce many functional secondary metabolites, including siderophores, cyclolipopeptides (CLPs), hydrogen cyanide, 2,4-diacetylphloroglucinol (DAPG), phenazine (PHZ), quinolones, and gluconic acid [50,51], and have been identified to have pathogenic or direct killing activity toward different RKNs among these secondary metabolites [45,46]. The *M. incognita*-toxic substance identified in the current study, 2-ethylhexan-1-ol, is a conventional basic chemical material that particularly serves as an important raw material for synthesizing esters or natural esters in the chemical engineering field. For example, it is widely used as an adjuvant for material synthesis and separation of metal ions (such as Cd^2+^) from complex solutions [52,53] or was widely used in food, medicine, cosmetics, and other chemical industries, with only one report with regard to the investigation of the physiological function of this substance in murine cells, which was found to be involved in peroxisome proliferation in both rat and mouse hepatocytes [54]. Only one report was found on nematicidal activity against the nematode [40]. Currently, no effort has been made to elucidate the action mechanism of this substance in the killing of *M. incognita*, namely, whether this substance kills *M. incognita* through a direct-action pattern, through indirect methods such as synergistic action with possible other low-content active substances, or by inducing nematodes to produce self-systemic lesions. Therefore, it is of particular interest to study the nematicidal activity and mechanism of action of chemically synthesized pure products of this substance, which is one of our next primary research goals.

## 4. Materials and Methods

### 4.1. Soil Sampling, Enrichment Culturing, and Isolation of Soilborne Bacteria

A total of 26 rhizosphere soil samples below the ground at approximately 10 cm were collected from vegetable (such as tomatoes and cucumbers) and fruit (such as kiwifruits and grapes) planting soils that were RKN-infected in different areas in China, and 10 g of each soil sample was initially added to 90 mL of NA medium (beef extract 3 g/L, peptone 10 g/L, NaCl 5 g/L, pH 7.0–7.2) for enrichment culturing for 24 h. Then, the culture suspension was diluted at a 10× gradient ratio and spread on NA plates, cultured at 28 °C for 24 h, picked into a single colony and streaked on NA plates to yield individual colonies. The individual morphology of these pure cultures of colonies was examined with a microscope to select those that were morphologically different isolates as the stock isolates. These isolates were generally prepared in glycerol tubes for initial storage at −80 °C for further use.

### 4.2. Bacterial Strains, Nematodes, Medium, and Culture Conditions

The wild-type strain *Brevundimonas bullata* MB756 is a newly isolated and identified bacterial isolate with nematocidal activity against the free-living model nematode *C.* *elegans* and the RKN *M. incognita* used in this study. *Escherichia coli* OP50 is a daily food for *C. elegans* larvae. The standard reference strain of the *E. coli* K12 strain used for G + C mol% determination was provided by CGSC (The Coli Genetic Stock Center) of Yale University (USA). The *C. elegans* N2 wild-type strain was provided by the *Caenorhabditis* Genetics Center (CGC) (College of Biological Sciences, University of Minnesota, MN 55108, USA), and it was routinely maintained at 20 °C on nematode growth medium (NGM) agar plates with *E. coli* OP50 as food [55]. The RKN *M. incognita* was routinely maintained by infecting the tomato plant *Lycopersicon esculentum* Mill cv. 144) in the laboratory greenhouse. *M. incognita* second-stage juveniles (J2) were collected for bioassays according to the previous descriptions by Manan et al. [34].

All bacterial cells were cultured in lysogeny broth (LB) medium (10.0 g/L tryptone, 5.0 g/L yeast extract, and 10.0 g/L NaCl, pH 7.0–7.2) for routine growth, unless otherwise specified. Other bacterial culture media, including nutrient broth medium (NBM) (beef extract 3 g/L, peptone 10 g/L, NaCl 5 g/L, pH 7.0–7.2), gelatin medium (MgSO_4_ 0.3 g/L, NaCl 0.3 g/L, K_2_HPO_4_ 0.3 g/L, agar 15 g/L, gelatin 10 g/L, pH 7.0-7.2), starch medium (peptone 10 g/L, beef extract 3 g/L, NaCl 5 g/L, agar 15 g/L, soluble starch 2 g/L, pH 7.4), peptone liquid medium (peptone 10 g/L, NaCl 5 g/L, pH 7.2–7.4), glucose peptone medium (glucose 5 g/L, peptone 5 g/L, NaCl 5 g/L, pH 7.2–7.4), Simmons’ citrate medium (sodium citrate 2 g/L, NaCl 5 g/L, MgSO4·7H_2_O 0.2 g/L, K_2_HPO_4_·3H_2_O 1 g/L, 1% thymol blue aqueous solution 10 mL, agar 15 g/L), ferric ammonium citrate medium (ferric ammonium citrate 0.5 g/L, sodium thiosulfate 0.5 g/L, beef extract 3 g/L, peptone 10 g/L, NaCl 5 g/L, agar 15 g/L, pH 7.4), and phenylalanine deaminase medium (yeast extract 3 g/L, NaCl 5 g/L, L-phenylalanine 1 g/L, Na_2_HPO_4_ 1 g/L, agar 15-20 g/L, pH 7.2–7.4), were used for bacterial isolation, morphological observation, and determinations of physiological and biochemical characteristics when appropriate.

### 4.3. Bacterial Isolate Identification

Various morphological observations under simple staining, Gram staining, and endospore staining, as well as physiological and biochemical tests, including catalase test, oxidase test, starch hydrolysis test, gelatin hydrolysis test, alanine deamination test, acetonyl methanol test (Voges-Proskauer test, V.P.), citrate utilization test, indole production test, methyl red (MR) test, and H_2_S production test, were all carried out according to the standard protocols described previously [56,57]. The growth curve of bacterial cells was generated following a common turbidimetric method [56]. Total bacterial DNA was prepared according to standard methods and purified by a mixture of phenol/chloroform/isoamyl alcohol [58], which was used for the amplification of the 16S rRNA gene fragment by polymerase chain reaction (PCR) from the genome of *B. bullata* MB756, using the primer pairs 27F: 5′−AGAGTTTGATCCTGGCTCAG−3′ and 1492R: 5′−GGTTACCTTGTTACGACTT−3′. The amplified products were sequenced and compared with the GenBank database of the National Center for Biotechnology Information (NCBI) database (https://blast.ncbi.nlm.nih.gov/Blast.cgi, accessed on 9 May 2022). Phylogenetic tree construction was performed using the software MEGA 5 [59] based on the neighbor-joining tree method [60]. The determination of G+C mol% of the strain MB756 was carried out by a UV–Vis spectrophotometer (DU-800 Nucleic Acids/Protein Analyzer, Beckman Coulter, Brea, CA, USA) according to the thermal denaturation method [61].

### 4.4. Screening of Nematode-Lethal Bacterial Strains

The initial screening of nematode-lethal bacterial strains from soil samples was performed based on 72-h cultured whole-cell suspensions. Basically, the isolated strains were inoculated in NA liquid medium and cultured with 200 r/min shaking at 28 °C for 72 h, and the normalized diluted cell suspension of each isolate at an optical density of 600 nm (OD_600_) 0.6 was shifted to the separate wells of 96-well microtiter plates (Costar, Corning Incorporated, Coming, NY, USA) for bioassays against *C. elegans* L4 stage larvae, following previously described procedures [24]. After an incubation at 20 °C for 5 days, larvae were examined for alive or dead status under an inverted fluorescence microscope (OLYMPUS IX73, Tokyo, Japan). Larvae were considered dead when they stopped moving and did not respond to a nudge with a platinum wire. The nematode mortality (%) and the corrected mortality (%) were calculated according to the following formulas. Bacterial strains with a corrected mortality greater than 50% were considered positive strains with respect to nematicidal activity.

(1) $$\mathrm{Mortality}\;(\%)=\frac{\mathrm{Number}\;\mathrm{of}\;\mathrm{dead}\;\mathrm{nematodes}}{\mathrm{Total}\;\mathrm{number}\;\mathrm{of}\;\mathrm{tested}\;\mathrm{nematodes}}\times \;100\%$$

(2) $$\mathrm{Corrected}\;\mathrm{mortality}(\%)=\frac{\mathrm{Mortality}\;\mathrm{of}\;\mathrm{treatments}\;-\;\mathrm{Mortality}\;\mathrm{of}\;\mathrm{controls}}{1\;-\;\mathrm{Mortality}\;\mathrm{of}\;\mathrm{controls}}\times \;100\%$$

The rescreening of nematicidal bacteria was performed using both *C. elegans* and *M. incognita* as the target nematodes to determine the corrected mortality of the culture supernatants of the initially screened strains. All parent strains were cultured in LB broth overnight and then inoculated into LB broth at 2% (vol/vol) inoculum size for 72 h of culture at 28 °C with 200 r/min shaking. The bacterial suspensions were harvested by centrifugation at 8000 r/min for 10 min and were then filtered through a microporous membrane (0.22 μm) to remove intact bacterial cells or debris. The filtrate was used as the SCS stock solution for bioassays against *M. incognita* following the procedures described previously [24]. The mortality and the corrected mortality were calculated according to Formulas (1) and (2). For MB756, cell density (OD_600_) and the corrected mortality against *M. incognita* and *C. elegans* were assayed using cell suspensions in different growth phases.

### 4.5. Assays on the Growth, Brood Size, and Motility of C. elegans

The SCS stock solution of *B. bullata* MB756 was diluted with phosphate-buffered saline (PBS, pH 7.2) to 0, 2×, 4×, 6× d, and 8× gradient diluent solutions. All nematodes were synchronized first, and the assays on the growth of *C. elegans* L1 stage larvae and on brood size and motility of L4 larvae *C. elegans* were carried out as previously described [34].

### 4.6. Bioassays of the SCS of MB756 under Different Treatments

For the treatments at different temperatures, five aliquots of the SCS stock solution of MB756 were incubated in 20 °C, 40 °C, 60 °C, 80 °C, and 100 °C water baths for 30 min and then quickly transferred to a 20 °C water bath for an additional 20-min incubation. The bioassays against *M. incognita* were performed following a previously described method [20]. The mortality and corrected mortality were calculated according to Formulas (1) and (2), respectively.

For treatments under different pH values, the SCS stock solution was adjusted to pH 3, 5, 7, 9, and 11, and then allowed to stand at room temperature (~25 °C). After 24 h, the pH of the all the test samples was adjusted to 7.0. pH was adjusted with 0.1 mol/L HCl or 0.1 mol/L NaOH. The bioassays were performed according to the abovementioned method.

The single colony of MB756 on an LB plate was removed and inoculated into another LB plate for culturing for 24 h and was further transferred to a successive new LB plate for a similar incubation and inoculation. The inoculations were performed up to 9 times. The bioassays against *M. incognita* were performed using the prepared SCS stock solutions from the MB756 cells that were transferred 1, 3, 5, 7, and 9 times, following the method described above.

For proteinase K treatment, the proteinase K-treated/untreated SCS stock solutions of MB756 were comparatively bioassayed as described above.

### 4.7. Organic Solvent Extraction of the SCS of MB756

Preparation of the SCS of MB756: a single colony of MB756 was obtained to inoculate into 5 mL of LB broth for culturing under 150 r/min shaking overnight at 28 °C. The cell inoculum was inoculated into 2.0 L of LB broth at 2% (vol/vol) inoculum size and cultured with 200 rpm shaking at 28 °C for 72 h. The cell suspensions were then centrifuged at 5000× *g* for 10 min at 4 °C to collect the supernatants, which were then filtered through disposable 0.22-μm microporous membrane filters (Millipore, Bedford, MA, USA) to remove cell debris.

Solvent extraction and bioassays: An equal volume of petroleum ether, chloroform, ethyl acetate, and N-butyl alcohol was added to each 20-mL aliquot of the SCS stock. After vigorous stirring in a separation funnel, the mixed solutions were centrifuged at 8000 rpm for 5 min. The organic phase of each mixture combination was carefully pipetted, and the extraction was repeated 3 times. The final organic phase of each extraction combination was collected and concentrated by an RE-2000A negative pressure rotary evaporator (Lichen Instrument Technology Co., Ltd., Shanghai, China) at 55 °C and −90 kPa to obtain dry solute matter. These solutes were redissolved with 2.0 mL of ddH_2_O, and bioassays against *M. incognita* were performed using the redissolved solute solutions of each combination following the previously described bioassay method [24]. The extraction with ddH_2_O was performed in parallel and was used as a control.

### 4.8. Purification and Identification of M. incognita-Toxic Substances of the MB756 SCS

Full-wavelength adsorption scanning: The extract solution prepared by ethyl acetate extraction underwent a full-wavelength scan from 190–900 nm with a UV–Vis spectrophotometer (DU800 nucleic acid/protein Analyzer, Beckman Coulter, Brea, CA, USA). The wavelength corresponding to the maximum adsorption peak was accepted as the maximum adsorption wavelength.

TLC of the extract of ethyl acetate extraction: Based on the pretests, a mixed solvent with chloroform and methanol at a 9:1 vol/vol ratio was selected as the mobile phase for the TLC assay. The extract of ethyl acetate was loaded on a Type C100008 TLC plate (Shanghai Haohong Biomedical Technology Co., Ltd., Shanghai, China) to be separated until the solvent layer approached the upper end of the plates. The plates were fumigated with iodine vapor for color development, and the detected zones were recorded and measured to calculate the *R_f_* value.

SGCC of the extract of ethyl acetate extraction: The ethyl acetate extract of the MB756 SCS was rotary-evaporated to obtain dry solute matter following the procedures described above. The dry matter was redissolved with a chloroform/methanol mixed solvent (9:1, vol/vol) and loaded into an SGCC equipped with a fraction collector (silica gel 200-300 mesh; column diameter 2.4 cm; filled column length 20.0 cm; Haiyang Chemical Co., Ltd., Qingdao, China). The chloroform/methanol mixed solution (9:1, vol/vol) was used as the eluent, and the detection wavelength was set as 257 nm. A total of 110 fraction tubes (2 mL of each tube) were collected following SGCC operation. All collected eluates corresponding to each adsorption peak were evaporated to obtain dry solute matter with a RE-2000A negative-pressure rotary evaporator at 55 °C and −90 kPa (Lichen Instrument Technology Co., Ltd., Shanghai, China). These solute matters were then redissolved in 2.0 mL of ddH_2_O, followed by bioassays against *M. incognita* as in the previous procedures [24], and the fraction with the highest corrected mortality against *M. incognita* was considered the main *M. incognita*-toxic substance for use in further analysis.

Preliminary identification of a main *M. incognita*-toxic substance by HPLC–MS: The collected fractions via SGCC with the highest corrected mortality were analyzed by high-resolution LC–MS (LC1290-QQQ-6470, Agilent, Japan) on a SinoPak C18 (5 μm × 4.6 mm × 250 mm) chromatographic column. The working conditions were set as follows: injection volume, 10 μL; column temperature, 30 °C; mobile phase, 40% methanol; flow rate, 1 mL/min; elution time, 20 min. Mass spectrometry acquisition parameters: MS2 scan, ESI^-^ mode; scanning range 100–1000 m/z; dry gas temperature 150 °C and flow rate 12 L/min; atomizer pressure 30 psi; sheath gas temperature 350 °C and flow rate 12 L/min, capillary voltage 3000 V; fragmentor, 135 V; and scan time 300 ms. Finally, ^1^H NMR was obtained with an INOVA 600 MHz NMR spectrometer (Varian, Palo Alto, CA, USA) using CDCl_3_ as the solvent to confirm the molecular structure. δH = 7.260 ppm with a proton frequency of 400 MHz (Figure S3). The molecular structure of the target substance was verified in silico using MestReNova 14.0 software (Mestrelab Research, Santiago de Compostela, Spain).

## 5. Conclusions

In summary, we performed a joint two-round screening of nematicidal bacteria from RKN-incident soils, which yielded a bacterial isolate with the highest corrected mortality against *C. elegans* and *M. incognita*. This strain was identified as *B. bullata* and named MB756. The SCS of MB756 exhibited not only high mortality against both nematodes over 86% but also detrimental effects on the growth, brood size, and motility of *C. elegans* larvae. The *M. incognita*-toxic substances in the SCS of the MB756 culture suspension were assayed for their tolerance capacity under heat, pH, and proteinase proteolysis conditions, and were then purified using TLC and SGCC. A main *M. incognita*-toxic substance was preliminarily identified as 2-ethylhexan-1-ol.

## Figures and Tables

**Table 1:** Nematode mortality of the extractions of the MB756 SCS against *M. incognita* using different organic solvents.

Extractants	Corrected Mortality (%) ^1^
Petroleum ether	42.89 ± 5.55
Chloroform phase	12.55 ± 6.62
Ethyl acetate phase	84.57 ± 8.33
n-butanol phase	44.41 ± 2.09
Aqueous phase	27.55 ± 4.27
MB756 SCS stock	88.27 ± 6.38

^1^ The mean ± SD (*n* = 3) was calculated based on three independent experiments.

## Supplementary Materials

The following supporting information can be downloaded at: https://www.mdpi.com/article/10.3390/pathogens11060708/s1, Figure S1: Bioassays of the supernatant of cell suspension (SCS) of 14 bacterial isolates with a corrected mortality (CM) higher than 50% against both M. incognita and C. elegans; Figure S2: Partial phenotypic characteristics of isolate No. 5-7. Figure S3: 1H NMR spectrum of the nematicidal compound of MB756.
