# Supplementary material for: Characterization of Nematicidal Activity and Nematode-Toxic Metabolites of a Soilborne Brevundimonas bullata Isolate
Source: Pathogens. 2022 Jun 20;11(6):708. doi: 10.3390/pathogens11060708 (PMC9229253; doi:10.3390/pathogens11060708)
Supplement: Supplementary file 1 [file pathogens-11-00708-s001.zip › pathogens-1744208-supplementary.pdf]

Supporting Material

Characterization of Nematicidal Activity and Nematode-toxic Metabolites of A Soilborne *Brevundimonas bullata* Isolate

Jiaoqing Li [1,†], Meijuan Ding [2,†], Xiaowen Sun [2], Zhe Li [2], Liangzheng Xu [1,*] and Lin Li [2,*]

[1] Guangdong Provincial Key Laboratory of Conservation and Precision Utilization of Characteristic Agricultural Resources in Mountainous Areas, School of Life Sciences, Jiaying University, Meizhou 514015, China; lijiaoqing@jyu.edu.cn.

[2] State Key Laboratory of Agricultural Microbiology, Huazhong Agricultural University, Wuhan 430070, China; dingmeijuan@webmail.hzau.edu.cn (M.D.); sunxiaowen525@webmail.hzau.edu.cn (X.S.); lizhe9913@163.com (Z.L.).

* Correspondence: lilin@mail.hzau.edu.cn (L.L.) and xlzheng@jyu.edu.cn (L.X.)

[†] These authors contributed equally to this paper.

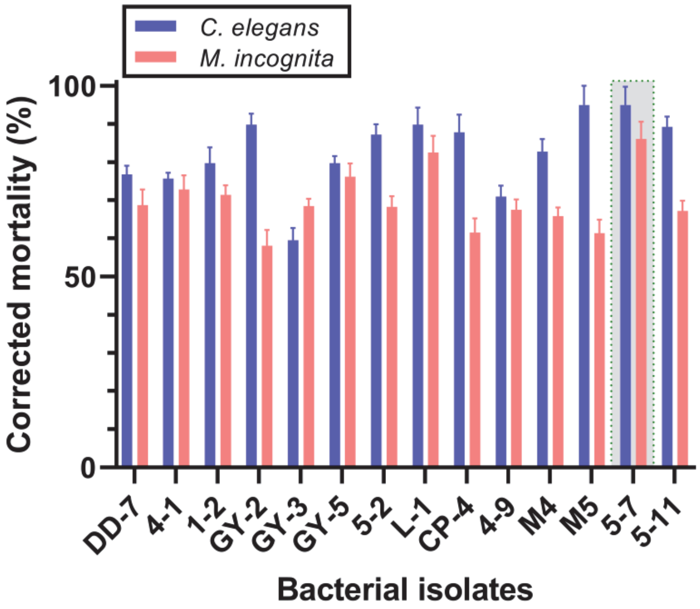

Figure S1. Bioassays of the supernatant of cell suspension (SCS) of 14 bacterial isolates with a corrected mortality (CM) higher than 50% against both *M. incognita* and *C. elegans*. All bacterial isolates were bioassayed for CM against *M. incognita* and *C. elegans* using the SCS cultured for 24 h in NBM broth, and their CM was the average mortality of three independent experiments and error bars represent the standard deviations from the means of these independent experiments. The strain shown in the dashed box (No. 5-7) that exhibited the highest CM against both nematodes, was selected as the parent strain for follow-up studies

A

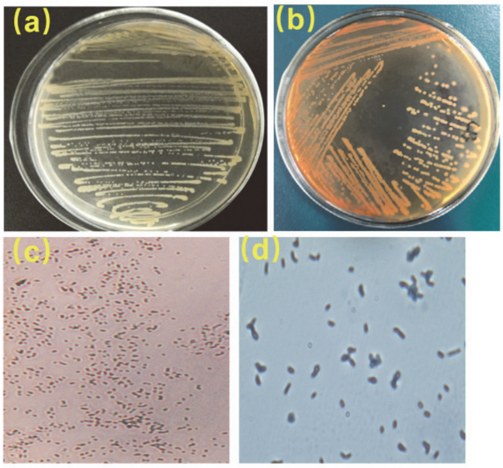

B

Characteristic	Isolate 5-7
Oxidase	+
Catalase	+
Indole production	–
Voges-Proskauer	–
M.R. test	–
H_2S production	–
Gelatin hydrolysis	–
Starch hydrolysis	–
Phenylalanine dehydrogenase	–
Citrate utilization	+
G + C mol%	67.6

Figure S2. Partial phenotypic characteristics of isolate No. 5-7. In A, (a), colonies grew on LB plate for 24 h; (b), colonies grew on LB plate for 3 days; (c), cells under Gram stain; (d), cells under spore stain. In B, +, positive; –, negative.

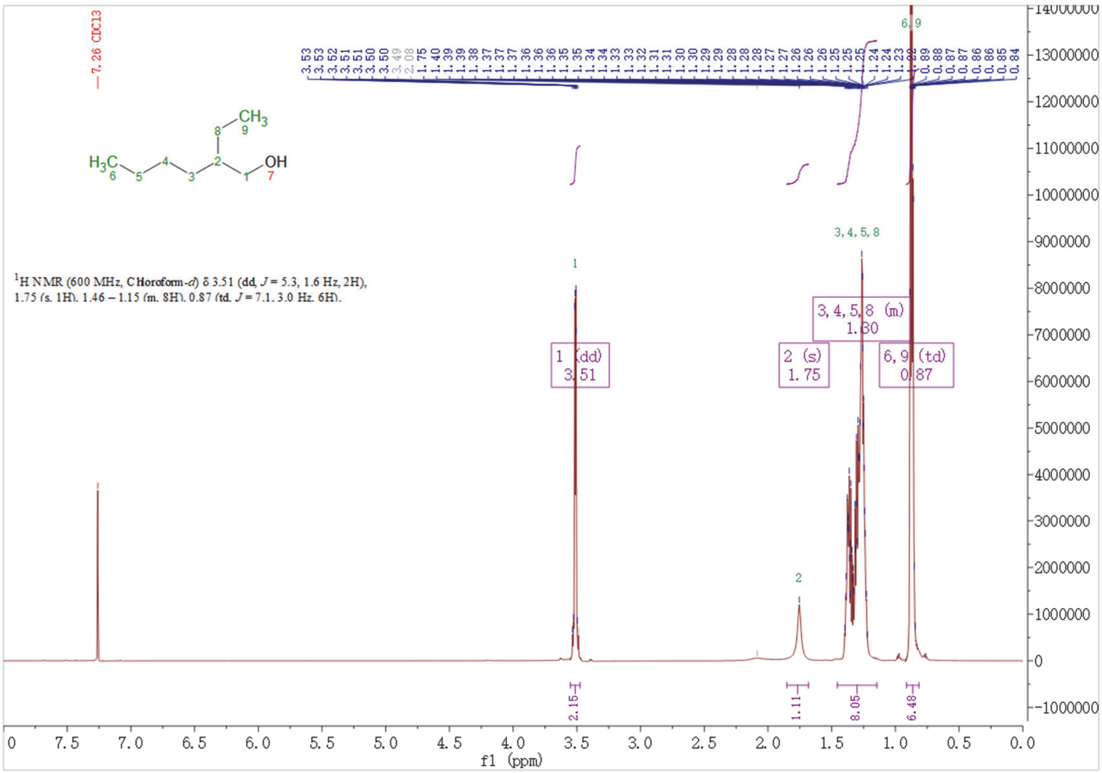

Figure S3. ¹H NMR spectrum of the nematicidal compound of MB756.
